# Systemic Sclerosis Presenting With Frank Exhibition of Mizutani's Sign

**DOI:** 10.7759/cureus.45387

**Published:** 2023-09-16

**Authors:** Samyak Ganjre, Bhushan Madke, Arjun Prakashey, Shivani Jangid, Sugat Jawade

**Affiliations:** 1 Dermatology, Venereology, and Leprosy, Jawaharlal Nehru Medical College, Datta Meghe Institute of Higher Education and Research, Wardha, IND; 2 Dermatology, Venereology, and Leprosy, All India Institute of Medical Sciences, Nagpur, IND

**Keywords:** mizutani's sign, systemic sclerosis, dermal fibrosis, autoimmune connective tissue disorders, clinical signs

## Abstract

Systemic sclerosis is an autoimmune connective tissue disease (AICTD) known for its hallmark feature of fibrosis affecting the skin, blood vessels, and viscera. The maintenance of the extracellular matrix (ECM) involves fibroblasts and other cells, which play a vital role in the degradation and replacement of damaged proteins such as collagens with new ones. The continuous stimulation of fibroblasts results in the overproduction of extracellular matrix proteins, leading to the progression of fibrosis.

Although the exact etiology of scleroderma is not clear, the onset of the condition has been attributed to genetic and environmental factors. Excessive collagen buildup in the dermis is the telltale sign of the disease. Clinicians face significant challenges in managing systemic sclerosis. Management is based on reducing or eliminating complaints to improve organ function, and frequently, multidisciplinary involvement is required. The aim of this case report is to emphasize the importance of the recognition of Mizutani's sign, which makes it prudent to rule out the presence of systemic sclerosis.

## Introduction

Systemic sclerosis or scleroderma is an autoimmune condition of unknown etiology, marked by features involving microvascular injury, immune dysregulation, and widespread fibrosis affecting multiple organs [1]. While cutaneous fibrosis is a feature most easily detectable in systemic sclerosis, the condition can also impact various other organs, including the lungs, heart, gastrointestinal tract, liver, and kidneys [2]. Although systemic sclerosis is classified as a rare disease, it has a high morbidity and mortality rate because approximately 50% of diagnosed patients succumb to multiorgan complications [3,4]. The risk of acquiring the disease is higher in females than males, with a ratio of 5:1 occurring in the age group 16-45 years [5]. Diagnosis is based on clinical features and investigations. Patients are managed to reduce or eliminate complaints and improve organ function, but this cannot cure them completely. Mizutani's sign refers to the loss of the peaked contour of the fingertips and replacement by a rounded contour. This was first specifically noted to be associated with patients of systemic sclerosis and hence warrants a complete and thorough dermatological and systemic examination to rule out or for the early diagnosis of systemic sclerosis [6].

## Case presentation

A 38-year-old female presented to the dermatology outpatient department (OPD) with complaints of taut skin over the face and the distal end of upper extremities, difficulty in swallowing solid foods, breathlessness, and dry cough. She also had complaints of joint pain in bilateral small and large joints, dryness of mouth, oral ulcers, difficulty in opening the mouth, and fissures along the angles of the mouth and was frequently constipated. On enquiring further, it was learned that she was a known case of systemic sclerosis but was irregular with her oral medications.

The patient initially learned about her disease by the change in the color of her fingers on exposure to cold about 12 years ago, for which she visited a clinician and was diagnosed with secondary Raynaud's phenomenon. Over the years, skin tightening involving the proximal areas of the forearms, lung involvement, and gut involvement manifested, and she was diagnosed with systemic sclerosis.

**Figure 1:**
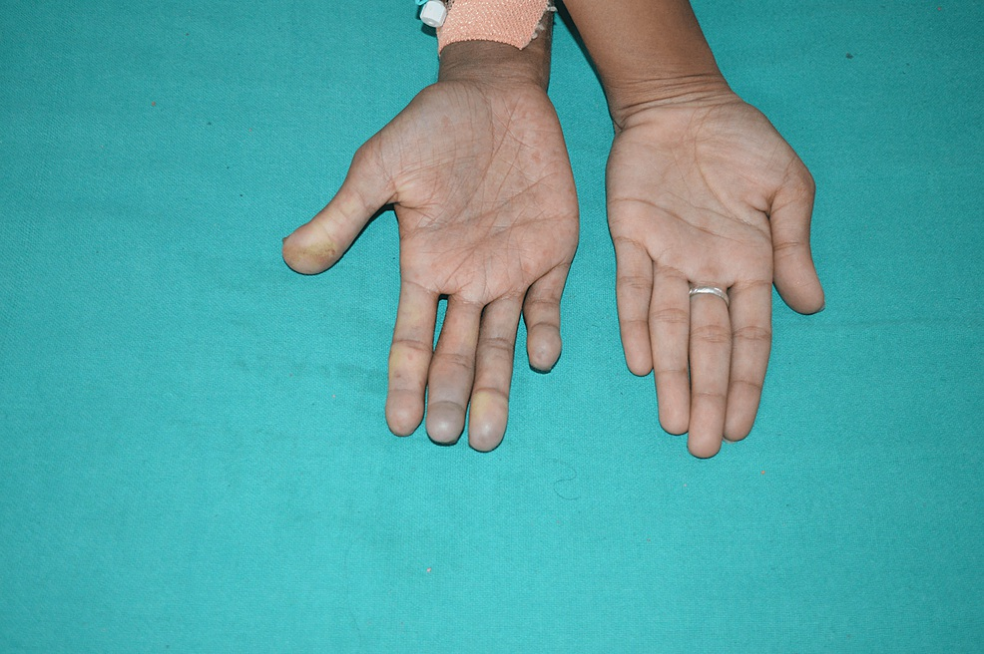
Mizutani's sign manifested in the right hand (palmar view) Peaked contour replaced by hemisphere-like contour (Mizutani's sign) on the third and fourth digits of the right hand

**Figure 2:**
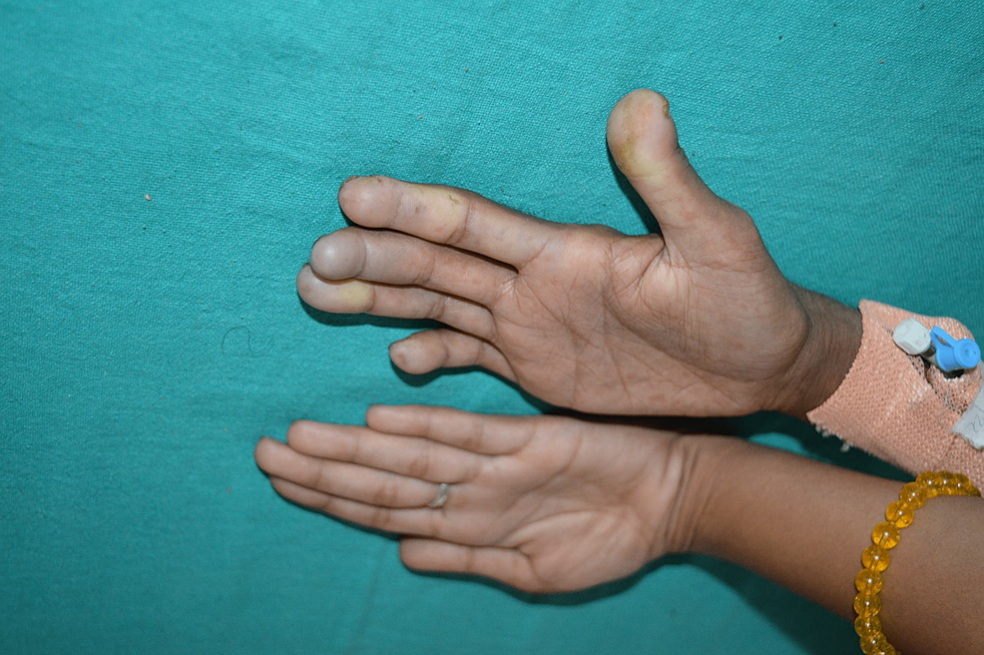
Mizutani's sign evident on the fingers of the right hand (oblique view) Mizutani's sign prominent on the third and fourth digits with a thickening of the fingers of the right hand

On clinical examination, Raynaud's phenomenon could be appreciated on exposure to cold. She had a modified Rodnan skin score (mRSS) of 2. On cutaneous examination, the skin of the face appeared taut and shiny with decreased forehead wrinkling. The nose had a characteristic parrot beak appearance, and the mouth had a purse string appearance. The skin was difficult to get hold of and felt like it was bound down; the fingers, specifically of her right hand, were swollen (sclerodactyly), and their tips had a hemispherical or rounded contour (Mizutani's sign) (Figure 1 and Figure 2).

Mouth opening was limited to two fingers, and the patient had multiple tooth caries. The patient was on oral antihypertensives (angiotensin-converting enzyme inhibitors). High-resolution computed tomography (HRCT) of the chest showed interstitial lung disease, whereas two-dimensional (2D) echocardiography showed grade 1 diastolic dysfunction. Mat-like telangiectasias were noted on the face and palms. Sclerodactyly could be appreciated. A striking rounded contour of her fingertips was noted.

## Discussion

The presence of the round fingerpad sign (Mizutani's sign) is commonly observed in individuals with Raynaud's phenomenon and sclerodactyly. In 1991, Mizutani and colleagues described the round fingerpad sign as an early indicator of scleroderma, highlighting its significance in the diagnosis of the condition [6]. Systemic sclerosis results in the fibrosis of the skin defined as excess deposition and the accumulation of extracellular matrix (ECM) in the dermis. The regulation of extracellular matrix levels in the tissue involves maintaining a delicate equilibrium between extracellular matrix synthesis, matrix metalloproteinase-mediated extracellular matrix degradation, and the inhibition of matrix metalloproteinase activity by tissue inhibitor of metalloproteinases (TIMP). An imbalance between matrix metalloproteinase and the tissue inhibitor of metalloproteinases can lead to the excess accumulation of collagen in the dermis. It is currently hypothesized that inflammation, autoimmune abnormalities, and vascular damage may trigger the activation of fibroblasts in systemic sclerosis. Consequently, abnormal fibroblasts in systemic sclerosis, responsible for fibrosis, may arise from a subset of these fibroblast cells that evade normal regulatory mechanisms [7].

The shape of the subcutaneous tissue plays a crucial role in determining the contour of the finger. In patients with scleroderma, dermal fibrosis occurs with the increased turnover of type III and VI collagens correlating with modified Rodnan skin score [6,8]. In progressive systemic sclerosis, dermal fibrosis significantly increases the surface tension of the skin more than the outward pressure exerted by the subcutaneous tissue, resulting in a reduction in the skin's surface area. Consequently, the normal peaked contour of the fingerpad is replaced by a hemisphere-like shape.

## Conclusions

Cutaneous signs are the hallmark of several autoimmune connective tissue diseases (AICTD). The presence of cutaneous signs can serve as a diagnostic clinical finding in many rheumatological disorders. Cutaneous signs such as the shawl sign, Gottron sign, Holster sign, and Ingram sign are well described in the dermatology literature.

The presence of a round fingerpad is highly suggestive of scleroderma. An astute clinician would suspect scleroderma and examine the patient for further systemic abnormalities. However, this sign is not exhibited in all patients of systemic sclerosis. Nevertheless, patients are advised to follow up regularly to keep in check the progression of the disease with regard to the involvement of the lungs and kidneys.
